# Shared and Unshared Feature Extraction in Major Depression During Music Listening Using Constrained Tensor Factorization

**DOI:** 10.3389/fnhum.2021.799288

**Published:** 2021-12-15

**Authors:** Xiulin Wang, Wenya Liu, Xiaoyu Wang, Zhen Mu, Jing Xu, Yi Chang, Qing Zhang, Jianlin Wu, Fengyu Cong

**Affiliations:** ^1^Department of Radiology, Affiliated Zhongshan Hospital of Dalian University, Dalian, China; ^2^School of Biomedical Engineering, Faculty of Electronic Information and Electrical Engineering, Dalian University of Technology, Dalian, China; ^3^Faculty of Information Technology, University of Jyväskylä, Jyvaskyla, Finland; ^4^Department of Psychology, College of Humanities and Social Sciences, Dalian Medical University, Dalian, China; ^5^Department of Neurology and Psychiatry, First Affiliated Hospital, Dalian Medical University, Dalian, China; ^6^School of Artificial Intelligence, Faculty of Electronic Information and Electrical Engineering, Dalian University of Technology, Dalian, China; ^7^Key Laboratory of Integrated Circuit and Biomedical Electronic System, Dalian University of Technology, Dalian, China

**Keywords:** CANDECOMP/PARAFAC, constrained tensor factorization, EEG, major depressive disorder, naturalistic music stimuli

## Abstract

Ongoing electroencephalography (EEG) signals are recorded as a mixture of stimulus-elicited EEG, spontaneous EEG and noises, which poses a huge challenge to current data analyzing techniques, especially when different groups of participants are expected to have common or highly correlated brain activities and some individual dynamics. In this study, we proposed a data-driven shared and unshared feature extraction framework based on nonnegative and coupled tensor factorization, which aims to conduct group-level analysis for the EEG signals from major depression disorder (MDD) patients and healthy controls (HC) when freely listening to music. Constrained tensor factorization not only preserves the multilinear structure of the data, but also considers the common and individual components between the data. The proposed framework, combined with music information retrieval, correlation analysis, and hierarchical clustering, facilitated the simultaneous extraction of shared and unshared spatio-temporal-spectral feature patterns between/in MDD and HC groups. Finally, we obtained two shared feature patterns between MDD and HC groups, and obtained totally three individual feature patterns from HC and MDD groups. The results showed that the MDD and HC groups triggered similar brain dynamics when listening to music, but at the same time, MDD patients also brought some changes in brain oscillatory network characteristics along with music perception. These changes may provide some basis for the clinical diagnosis and the treatment of MDD patients.

## 1. Introduction

Major depressive disorder (MDD) is a globally prevalent mental disorder with multifactorial causes (Belmaker and Agam, 2008; Gotlib and Joormann, 2010; Jia et al., 2010). Over the past decades, the neural mechanisms of MDD have been widely explored using non-invasive neuroimaging techniques, like functional magnetic resonance imaging (fMRI), electroencephalogram (EEG), and magnetoencephalography (Gotlib and Hamilton, 2008; Kaiser et al., 2015). Most previous studies of MDD are under the conditions of resting states or well-controlled stimuli. In recent years, naturalistic paradigms have been challenging conventional paradigms because they can approximate real-life experiences using naturalistic and continuous stimuli, like music listening and movie watching (Hasson et al., 2004; Sonkusare et al., 2019). Naturalistic paradigms have shown a clinical potential in mental disorders, such as MDD, autism-spectrum disorder, paranoia, and so on (Sonkusare et al., 2019). Music perception can induce emotional arousal for affective processing, and music therapy has shown the feasibility in MDD treatment (Michael et al., 2005; Maratos et al., 2008). However, few studies have correlated the music perceptive arousal with neural mechanisms in MDD, and the studies mainly explored the networks of brain connectivity at the source space (Liu et al., 2020, 2021; Zhu et al., 2021). Furthermore, the current findings are often inconsistent or even contradictory due to the different methodological approaches and involved participants, unified neural mechanisms of MDD (in music perception) can not be concluded (Zhi et al., 2018). Therefore, it is still urgent and important to develop novel experimental designs and advanced computational methodologies to better investigate the neural biomarkers of MDD. In our study, we aim to investigate the biomarkers in MDD during music listening using EEG data at the sensor level.

Due to the high temporal resolution, electroencephalography (EEG) signals contain rich spectral contents. During continuous cognition, the spatial reconfiguration will be dynamically sustained along time, and the spatial signatures are modulated by oscillations (Buzsaki, 2006; Yan et al., 2020; Sadaghiani et al., 2021). Apparently, the EEG signals can be represented by the high-order multi-way array, i.e., tensor, which can fully describe the inherent interaction relationships among multiple dimensions in the data (Kolda and Bader, 2009; Cichocki et al., 2015; Cong et al., 2015). Recently, some studies have investigated the electrophysiological signatures characterized by spatio-temporal-spectral modes of covariation from the tensor representation of EEG data via Canonical Polyadic (CP) decomposition (Mørup et al., 2007; Cong et al., 2012, 2015; Zhu et al., 2020). These studies are based on the assumption of spatial-, spectral-, temporal consistency, which means that each subject or group shares the same frequency-specific brain topography or networks with the same temporal dynamics (Cong et al., 2012, 2015). However, except for common features, individual features should also be considered for subject or group differences (Wang et al., 2020; Liu et al., 2021). Therefore, the incomplete spatial, temporal and spectral consistency should be assumed to better fit the data characteristics and practical applications. Meanwhile, numerous versions of independent component analysis (ICA)-based methods and their group analysis variants have also been popularly adopted to analyse EEG signals (Cong et al., 2013; Labounek et al., 2018; Zhu et al., 2021). For example, Zhu et al. performed group-level spatial Fourier ICA to explore the frequency-specific brain networks of musical feature processing, and found the alpha lateral component engaged in music perception in MDD (Zhu et al., 2021). These two-way methods simply stack or concatenate the extra modes of EEG signals into two-way matrix for processing, but lose the potential internal relationships among modes and destroy the inherent multi-linear structure of the data (Cong et al., 2015). Considering the high-dimensional structure of the data and the incomplete consistency of different modes, we applied a constrained tensor factorization model by imposing nonnegative and coupled constraints in the present study, by which we can access to the shared and unshared features simultaneously. Liu et al. only considered the coupling structure in spectral and connectivity modes and explored the connectivity alteration in EEG signals during music perception in MDD using tensor decomposition-based methods (Liu et al., 2021). Therefore, different from the previous work (Liu et al., 2020, 2021; Zhu et al., 2021), we further consider the coupling characteristics in the temporal modes between the MDD and healthy controls (HC) data at the sensor-level, i.e., we assume that some of the spatio-temporal-spectral patterns are the same between the two group data while the rest are different.

In this study, for the EEG signals of MDD and HC groups during music listening, we investigated spatio-temporal-spectral modes of covariation using a coupled nonnegative tensor factorization framework, aiming to extract the shared and unshared features between/in the two groups. Specifically, we first recorded the EEG signals during freely listening to a piece of 512-s tango music. Using the time-frequency representation, we then constructed two fourth-order tensors of time, frequency, space and participant for the two groups. Considering the incomplete consistency in spatio-temporal-spectral modes, we applied the triple-coupled nonnegative tensor factorization model optimized by alternating direction method of multipliers (ADMM, Boyd et al., 2011) algorithm, which enables the simultaneous decomposition of shared components and unshared components among tensors. Meanwhile, we extracted five long-term musical features from the musical stimulus using musical information retrieval in order to build the connections with the extracted components from EEG signals. Next, correlation analysis was performed between temporal courses of musical features and the extracted temporal components from EEG signals, and we obtained the spatio-temporal-spectral brain dynamics of interest that were believed to be activated by music modulation. Following this, hierarchical clustering was conducted on the shared and unshared spatial components of the results from multiple runs. Finally, we obtained two clusters of feature patterns shared by MDD and HC groups, as well as one cluster from the HC group and two clusters from the MDD group, which may contribute to the biomarkers for the clinical diagnosis and treatment for MDD patients. The proposed framework based on the constrained tensor factorization is completely data-driven and provides a solution to extract the shared and unshared spatio-temporal-spectral features of EEG signals from different groups.

## 2. Materials and Methods

### 2.1. Data Description

#### 2.1.1. Participants

In this study, we analyzed the data from 39 participants in total, including nineteen healthy controls (HC) and 20 major depression disorder (MDD) patients. No one was reported to have hearing loss and formal training in music. The mental health of each participant was evaluated and diagnosed by a clinical expert using Hamilton Rating Scale for Depression (HRSD), Hamilton Anxiety Rating Scale (HAMA) and Mini-Mental State Examination (MMSE). The relative values of these indices as well as age, gender, education, duration of illness for HC and MDD groups are listed in Table 1. All participants signed the informed consent forms approved by the ethics committee of First Affiliated Hospital of Dalian Medical University and Dalian University of Technology.

**Table 1:** Basic information of the participants in HC and MDD groups.

	**HC group**	**MDD group**	**HC vs. MDD**
	**Mean ± SD**	**Mean±SD**	**p-value**
Age (years)	38.4 ± 11.8	42.9 ± 11.0	>0.05^*^
Gender (F:M)	14:5	14:6	>0.05^**^
Education (years)	13.6 ± 3.8	12.8 ± 3.4	>0.05^*^
Duration (months)	-	12.8 ± 8.5	-
HRSD	2.4 ± 1.3	23.3 ± 3.6	<0.01^*^
HAMA	2.4 ± 1.3	19.2 ± 3.0	<0.01^*^
MMSE	28.2 ± 0.9	28.1 ± 1.1	>0.05^*^

* *The p-value is calculated via t-test*.

** *The p-value is calculated via chi-squared test. Duration is the duration of illness*.

*HC, healthy controls; MDD, major depression disorder patients; F, Female; M, Male; NRSD, Hamilton Rating Scale for Depression; HAMA, Hamilton Anxiety Rating Scale; MMSE, Mini-Mental State Examination*.

#### 2.1.2. EEG data

A 512-second modern tango music “Adios Nonino” played by Astor Piazzolla was adopted as the naturalistic stimulus in this experiment. The participants were told to seat as still as possible with eyes open and listen to the tango music. The ongoing EEG data were recorded using the international 10–20 system-based Neuroscan Quik-cap device of 64 electrodes with the sampling frequency of 1,000 Hz. The recorded EEG data were preprocessed off-line using MATLAB software and EEGLAB toolbox (Delorme and Makeig, 2004), down-sampled to 256 Hz, and filtered by the high-pass and low-pass filters with 4 Hz and 30 Hz cut-off frequencies. The components indicating eye movements artifacts were rejected by independent component analysis (ICA). The data were also visually checked to remove the obvious artifacts brought by head movement, and then used for further analysis.

#### 2.1.3. Musical Features

In this study, five long-term musical features (including two tonal and three rhythmic features) were extracted by a frame-by-frame analysis method using MIR toolbox (Lartillot and Toiviainen, 2007). The duration of each frame was 3 s and the overlap ratio between two frames was 66.7%, which was consistent with the window settings in the time-frequency representation of EEG data. Finally, in order to match the length of recorded EEG data, we selected the first 500 samples for each time course of these musical features with a 1 Hz sampling rate. For the tonal features, Mode denotes the strength of major or minor mode, and Key Clarity is the measure of the tonal clarity. For the rhythmic features, Fluctuation Centroid is defined as the geometric mean of the fluctuation spectrum, and it represents the global repartition of rhythm periodicities within the range of 0~10 Hz, indicating the average frequency of these periodicities. Fluctuation entropy is the Shannon entropy of the fluctuation spectrum, and it represents the global repartition of rhythm periodicities. Pulse Clarity is regarded as an estimate of clarity of the pulse. The details of musical features and extraction method can be found in the previous studies (Alluri et al., 2012; Cong et al., 2013).

### 2.2. Constrained Tensor Factorization

#### 2.2.1. Tensor Construction

In order to comprehensively analyze the data from more aspects, we first converted the data from the time domain to the time-frequency domain. Specifically, we obtained the time-frequency representation of the preprocessed EEG data via performing the short-time Fourier transform (STFT) on the time series of each channel for each participant. The Hamming window was adopted as the window function, with the window length of 3 s and 66.7% overlap ratio between windows. The number of Fourier points in each window was 1,280, which was five times of the sampling rate. Finally, for the data of each channel, we obtained the spectrograms with the size of 130 (frequency bins) × 500 (time samples). Therefore, for the HC group and MDD group, the EEG data were reconstructed to two fourth-order tensors with the dimensions of channel (64), frequency (130), time (500) and participants (19 or 20), i.e., $X_{\text{HC}}\in {\mathbb{R}}_{+}^{64\times 130\times 500\times 19}$ and $X_{\text{MDD}}\in {\mathbb{R}}_{+}^{64\times 130\times 500\times 20}$.

#### 2.2.2. Tensor Factorization

Tensors, also known as multi-way arrays, are the higher-order generalizations of scalars, vectors and matrices. So far, the two most commonly used models for tensor factorization are the canonical polyadic [CP (Hitchcock, 1927), also known as CANDECOMP/PARAFAC (Carroll and Chang, 1970; Harshman, 1970)] model and the Tucker model (Tucker, 1966). The CP model, as a special case of the Tucker model, has better unique identifiability under mild conditions, and was adopted in this study. Thus, the factorizations of the data $X_{\text{HC}}$ and $X_{\text{MDD}}$ can be, respectively expressed, using a sum of fourth-order rank-one tensors or a set of factor matrices, as


(1) $$X_{\text{HC}}\approx \sum_{r\;=\;1}^{R_{\text{HC}}}a_{r}^{\left(1\right)}○a_{r}^{\left(2\right)}○a_{r}^{\left(3\right)}○a_{r}^{\left(4\right)}=〚A_{1},A_{2},A_{3},A_{4}〛$$

and


(2) $$X_{\text{MDD}}\;\approx \;\sum_{r\;=\;1}^{R_{\text{MDD}}}b_{r}^{\left(1\right)}○b_{r}^{\left(2\right)}○b_{r}^{\left(3\right)}○b_{r}^{\left(4\right)}\;=〚B_{1},B_{2},B_{3},B_{4}〛$$

where *R*_HC_ and *R*_MDD_ are the tensor rank or the number of components that will be extracted from $X_{\text{HC}}$ and $X_{\text{MDD}}$. “○” denotes the vector outer product. $A_{i}=\left[a_{1}^{\left(i\right)}\cdots a_{R_{\text{HC}}}^{\left(i\right)}\right]\in {\mathbb{R}}_{+}^{I_{i}\times R_{\text{HC}}}$ and $B_{i}=\left[b_{1}^{\left(i\right)}\cdots b_{R_{\text{MDD}}}^{\left(i\right)}\right]\in {\mathbb{R}}_{+}^{J_{i}\times R_{\text{MDD}}}$, *i* = 1, 2, 3, 4, correspond to the factor matrices in the spatial, spectral, temporal and participant modes, respectively.

#### 2.2.3. Non-negative Coupled Tensor Factorization

Constrained tensor factorization can accurately extract and explain the hidden components from the input data, by imposing particular penalties/regularizations (e.g., nonnegativity, sparsity, smoothness, coupling) on the corresponding factor matrices in the factorization process. Consider the connections and inherent characteristics of the data $X_{\text{HC}}$ and $X_{\text{MDD}}$, we imposed the nonnegative constraint in all of modes and the coupling structure in spatial, spectral, temporal modes across the data during the optimization. For the factor matrices ***A***_*i*_ and ***B***_*i*_, *i* = 1⋯4, we assume each one consists of two parts as $A_{i}=\left[A_{i}^{C}A_{i}^{I}\right]$ and $B_{i}=\left[B_{i}^{C}B_{i}^{I}\right]$, where $A_{i}^{C}=B_{i}^{C}\in {\mathbb{R}}_{+}^{I_{i}\times L_{i}}$ denotes *L*_*i*_ components are shared by the data, while $A_{i}^{I}\in {\mathbb{R}}_{+}^{I_{i}\times \left(R_{\text{HC}}-L_{i}\right)}$ and $B_{i}^{I}\in {\mathbb{R}}_{+}^{I_{i}\times \left(R_{\text{MDD}}-L_{i}\right)}$ correspond to the individual components in each data. *L*_*i*_ is the number of shared components among data (*L*_4_ = 0). Thus, the shared and unshared components can be simultaneously extracted via the formulated nonnegative coupled tensor factorization (NCTF) model, in which the objective function should be minimized as follows:


(3) $$\begin{array}{l} \text{minmize }L(A_{i},B_{i})={\left\|X_{\text{HC}}-〚A_{1},A_{2},A_{3},A_{4}〛\right\|}_{F}^{2}+\|X_{\text{MDD}}\\ {\;-〚B_{1},B_{2},B_{3},B_{4}〛\|}_{F}^{2} \end{array}$$

$$\text{subject to }A_{i}\geq 0,B_{i}\geq 0\text{ for }i=1\cdots 4$$

where ||·||_*F*_ denotes the Frobenius norm.

#### 2.2.4. Algorithm Optimization

Obviously, the minimization in Equation (3) is not convex but in fact an NP-hard problem. Aiming for an easy-to-handle and robust approximation, we propose to use the ADMM method within the framework of block coordinate descent (BCD) to solve the above optimization problem, which has been proven to be very efficient in the regularized matrix and tensor factorizations (Huang et al., 2016; Schenker et al., 2020). Specifically, BCD framework can obtain a local solution of Equation (3) by converting it into a set of subproblems, in which the factor matrices ***A***_1_&***B***_1_, ***A***_2_&***B***_2_, ***A***_3_&***B***_3_ and ***A***_4_&***B***_4_ will be updated alternatively one by one in each iteration, then each subproblem can be solved using ADMM strategy. Taking the update of the primal variable pair ***A***_*i*_ and ***B***_*i*_, *i* = 1, ⋯4 as an example, the problem in Equation (3) can be reformulated by introducing the auxiliary variables ${\tilde{A}}_{i}$ and ${\tilde{B}}_{i}$ as follows:


(4) $$\begin{array}{l} \text{minmize }L(A_{i},{\tilde{A}}_{i},B_{i},{\tilde{B}}_{i})={\left\|X_{\text{HC}}-〚A_{1},A_{2},A_{3},A_{4}〛\right\|}_{F}^{2}\\ \;+{\left\|X_{\text{MDD}}-〚B_{1},B_{2},B_{3},B_{4}〛\right\|}_{F}^{2} \end{array}$$

$$\text{subject to }A_{i}={\tilde{A}}_{i},B_{i}={\tilde{B}}_{i},\;{\tilde{A}}_{i}\geq 0\;{\tilde{B}}_{i}\geq 0.$$

The augmented Lagrangian function of Equation (4) is given as:


(5) $$\begin{array}{l} \text{minmize }L(A_{i},\tilde{A_{i}},\Lambda_{{\text{A}}_{i}},B_{i},\tilde{B_{i}},\Lambda_{{\text{B}}_{i}})\;={\left\|X_{\text{HC}}-〚A_{1},A_{2},A_{3},A_{4}〛\right\|}_{F}^{2}\\ \;+\;\rho_{i}{\left\|A_{i}\;-\;{\tilde{A}}_{i}\;+\;\Lambda_{i}\right\|}_{F}^{2}\;+\;{\left\|X_{\text{MDD}}-〚B_{1},B_{2},B_{3},B_{4}〛\right\|}_{F}^{2}\\ \;+\;\sigma_{i}{\left\|B_{i}\;-\;{\tilde{B}}_{i}\;+\;\Gamma_{i}\right\|}_{F}^{2} \end{array}$$

where $\Lambda_{i}\in {\mathbb{R}}_{+}^{I_{i}\times R_{\text{HC}}}$ and $\Gamma_{i}\in {\mathbb{R}}_{+}^{J_{i}\times R_{\text{MDD}}}$ are the Lagrangian multipliers or dual variables, ρ_*i*_ and σ_*i*_ are the penalty parameters. The solutions for Equation (5) can be calculated by successively minimizing L with respect to ***A***_*i*_, ***B***_*i*_, ${\tilde{A}}_{i}$, ${\tilde{B}}_{i}$, **Λ**_*i*_ and **Γ**_*i*_ one at a time while fixing the others until convergence. The update rules of these variables can be seen in Equation (6), where ***F***_*A*_ = ***A***_4_⊙⋯***A***_*i*+1_⊙⋯***A***_*i*−1_⊙***A***_1_, and ***F***_*B*_ = ***B***_4_⊙⋯***B***_*i*+1_⊙⋯***B***_*i*−1_⊙***B***_1_, “⊙” is the Khatri-Rao product. $X_{\text{HC},i}\in {\mathbb{R}}_{+}^{I_{i}\times \prod_{k\;\neq \;i}^{4}}$ and $X_{\text{MDD},i}\in {\mathbb{R}}_{+}^{J_{i}\times \prod_{k\;\neq \;i}^{4}}$ mean the mode-*i* matricization of tensors $X_{\text{HC}}$ and $X_{\text{MDD}}$. (·)^*C*^ represents the first *L*_*i*_ columns of the matrix and (·)^*I*^ is the remaining columns. Analogously, we can obtain the updating solutions of other variables. It should be noted that the derivation of the coupling parts $A_{i}^{C}=B_{i}^{C},i=1,2,3$ should be refer to the information from $X_{\text{HC}}$ and $X_{\text{MDD}}$. The entire optimization process is termed as NCTF-ADMM algorithm and summarized in Algorithm 1. Moreover, in this study, two stopping criteria were adopted in the algorithm optimization. (i) ||RelErr_*new*_ − RelErr_*old*_|| < *tol*, it means that the relative error (RelErr) change of data fittings between the adjacent iterations should be smaller than *tol* (here we set *tol* = 10*e* − 8). RelErr is defined as $\text{RelErr}=\frac{||X_{\text{HC}}-{\tilde{X}}_{\text{HC}}|{|}_{F}}{||X_{\text{HC}}|{|}_{F}}+\frac{||X_{\text{MDD}}-{\tilde{X}}_{\text{MDD}}|{|}_{F}}{||X_{\text{MDD}}|{|}_{F}}$, ${\tilde{X}}_{\text{HC}}$ and ${\tilde{X}}_{\text{MDD}}$ are the recovered tensors. Meanwhile, the Fit value is defined as $\text{Fit}=1-\frac{\text{RelErr}}{2}$. (ii) The maximum number of iterations is no more than 1,000.

**Algorithm 1:**
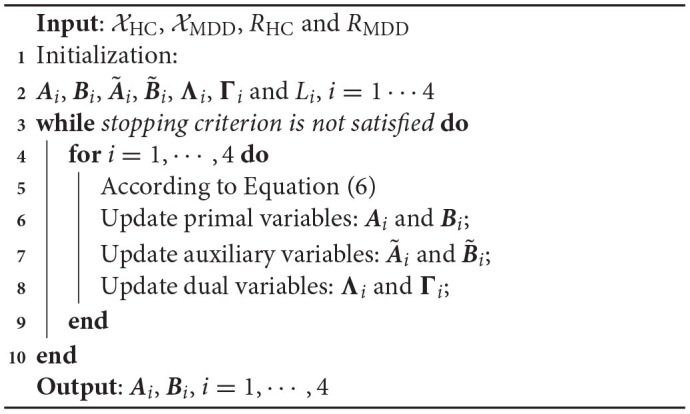
NCTF-ADMM algorithm.

### 2.3. Correlation Analysis

To discover the relationships between musical stimuli and the EEG data, five musical features were first extracted from the music stimuli. After performing NCTF-ADMM algorithm on the HC and MDD tensor data, a correlation analysis method was conducted between time courses of extracted temporal components and time courses of musical features. We adopted Pearson correlation to calculated the correlation efficient, and applied the Monte Carlo method and permutation test to determine the significant thresholds of the correlation and correct for multiple comparisons (Alluri et al., 2012; Cong et al., 2013; Wang et al., 2020). For the time course of each musical feature, a threshold of correlation coefficient at a significant level of *p* < 0.05 was calculated with the time courses of extracted temporal components. Then those components whose temporal components satisfied significant correlation were considered to be related to musical stimuli, and will be of interest and further analyzed.


(6) $$\{\begin{array}{l} A_{i}^{C}=B_{i}^{C}=\\ \;\left[X_{\text{HC},i}F_{A}^{C}\;+\;X_{\text{MDD},i}F_{B}^{C}\;-\;A_{1}^{I}{\left(F_{A}^{I}\right)}^{T}F_{A}^{C}\;-\;B_{1}^{I}{\left(F_{B}^{I}\right)}^{T}\;F_{B}^{C}-\Lambda_{i}^{C}-\Gamma_{i}^{C}+\rho_{i}{\tilde{A}}_{i}^{C}+\sigma_{i}{\tilde{B}}_{i}^{C}\right]\\ \;{\left[{\left(F_{A}^{C}\right)}^{T}F_{A}^{C}+{\left(F_{B}^{C}\right)}^{T}F_{B}^{C}+\left(\rho_{i}+\sigma_{i}\right)I\right]}^{-1}\\ A_{i}^{I}=\left[X_{\text{HC},i}F_{A}^{I}-A_{i}^{C}{\left(F_{A}^{I}\right)}^{T}F_{A}^{C}-\Lambda_{i}^{I}+\rho_{i}{\tilde{A}}_{1}^{I}\right]{\left[{\left(F_{\text{A}}^{I}\right)}^{T}F_{\text{A}}^{I}+\rho_{i}I\right]}^{-1}\\ B_{i}^{I}=\left[X_{\text{MDD},i}F_{B}^{I}-B_{i}^{C}{\left(B_{A}^{I}\right)}^{T}F_{B}^{C}-\Gamma_{i}^{I}+\sigma_{i}{\tilde{B}}_{i}^{I}\right]{\left[{\left(F_{\text{B}}^{I}\right)}^{T}F_{\text{B}}^{I}+\sigma_{i}I\right]}^{-1}\\ {\tilde{A}}_{i}={\left[A_{i}+\Lambda_{i}\right]}_{+},\;{\tilde{B}}_{i}={\left[B_{i}+\Gamma_{i}\right]}_{+},\;\Lambda_{i}=\Lambda_{i}+A_{i}-{\tilde{A}}_{i},\;\Gamma_{i}=\Gamma_{i}+B_{i}-{\tilde{B}}_{i} \end{array}$$

### 2.4. Shared and Unshared Feature Clustering

In order to guarantee the reliability of the results, we independently performed the constrained tensor factorization multiple times (in this study we set 50 times). After performing correlation analysis for the multiple results, we adopted clustering method to cluster the shared and unshared spatial components, respectively. Meanwhile, we merged the corresponding spectral component and counted the musical feature distributions that were involved in the same cluster. For stable clustering, we adopted hierarchical agglomerative clustering algorithm, in which complete linkage was used to calculate the furthest distance (here we used correlation) between pairs of clusters and the pairs of clusters with the nearest distance were merged.

## 3. Results

The EEG data used in this study can be obtained from the corresponding authors according to reasonable requirements, and the code to reproduce the simulation in Section 3.1 is available at https://github.com/xiulinwang/FrontierHN-NCTF-ADMM.

The following experiments are done with the following computer configurations; CPU: Intel^Ⓡ^ Xeon(R) E5-2680 v2 @ 2.80 Hz × 40; Memory: 125.80 GiB; System: 64-bit ubuntu 16.04; Matlab R2014b.

### 3.1. Simulation Results

**Figure 1:**
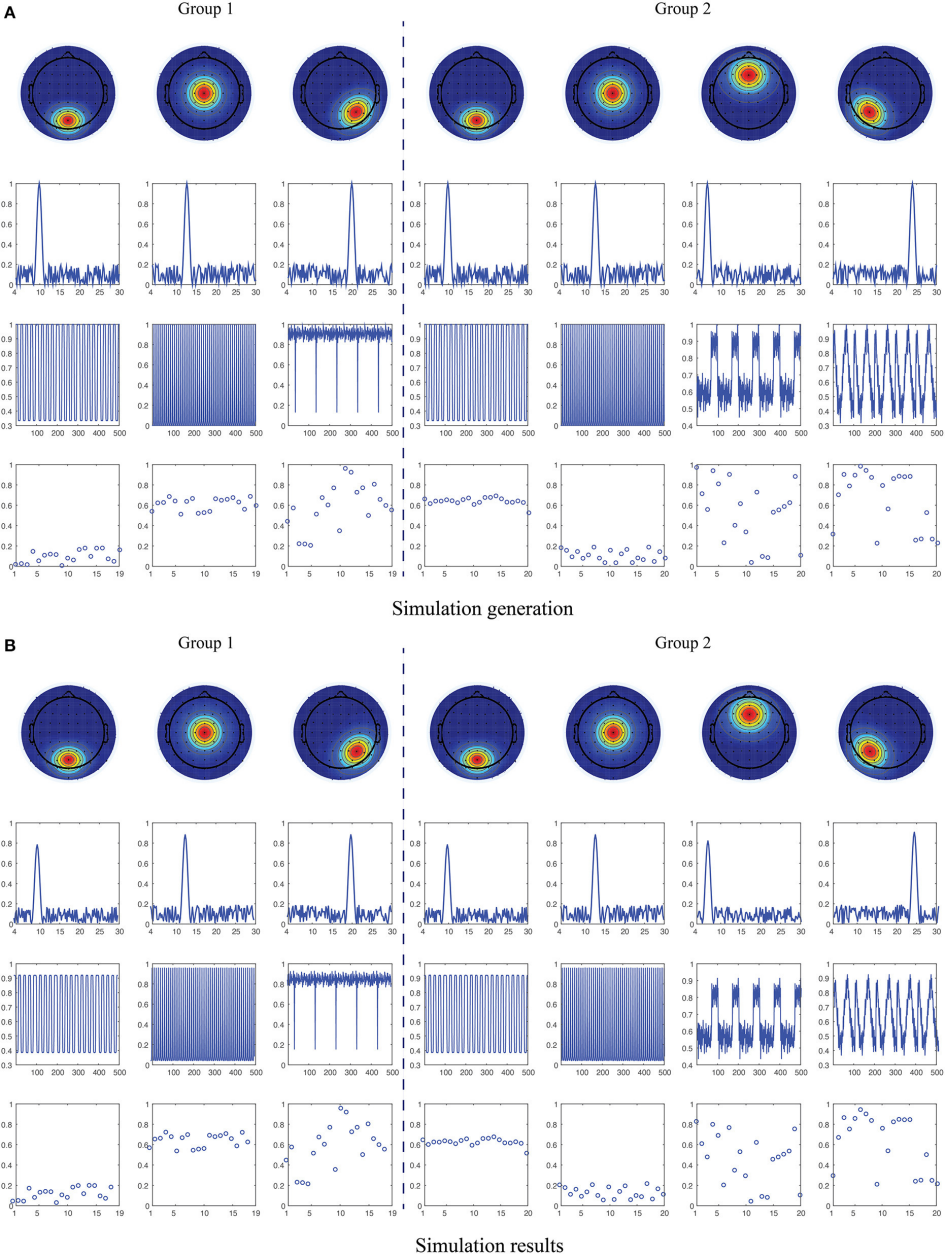
Illustration of simulation generation and recovered results. **(A)** Simulated spatial, spectral, temporal and participant patterns (from top to bottom) for the two groups with partially coupled constraints in the first two components of spatial, spectral and temporal modes (see them in the 1st, 2nd, 4th, and 5th columns). **(B)** Reconstructed spatial, spectral, temporal and participant patterns (from top to bottom) using constrained tensor factorization methods.

In this study, we first designed the simulation data to verify the performance of the proposed constrained tensor factorization method. We generated four kinds of predefined component factors indicating spatial, spectral, temporal and participant information, respectively, and then constructed two fourth-order tensors representing the simulated HC and MDD data via the outer product of corresponding vectors as follows:


(7) $$X=\sum_{r\;=\;1}^{R}u_{r}^{\left(1\right)}u_{r}^{\left(2\right)}u_{r}^{\left(3\right)}u_{r}^{\left(4\right)}$$

where $u_{r}^{\left(1\right)}\in {\mathbb{R}}_{+}^{64\times 1}$, $u_{r}^{\left(2\right)}\in {\mathbb{R}}_{+}^{130\times 1}$, $u_{r}^{\left(3\right)}\in {\mathbb{R}}_{+}^{500\times 1}$ and $u_{r}^{\left(4\right)}\in {\mathbb{R}}_{+}^{19\left(20\right)\times 1}$ present topography, power spectrum, waveform and magnitude of participant, respectively, as shown in Figure 1A. $X\in {\mathbb{R}}_{+}^{64\times 130\times 500\times 19\left(20\right)}$ denotes the ground true EEG data, and the noised synthetic EEG data was generated as


(8) $$Z=\sigma_{x}\frac{X}{\left\|X\right\|}+\sigma_{n}\frac{N}{\left\|N\right\|}$$

where σ_*x*_ and σ_*n*_ denote the levels of signal and noise. N is the noise tensor data uniformly distributed on the open interval (0, 1) and of the same size with X. SNR refers to the signal-to-noise ratio defined as SNR = 10log10(σ_*x*_/σ_*n*_), and we set SNR to 20dB in this experiment. For the two synthetic tensors, the number of component is set to *R*_HC_ = 3 and *R*_MDD_ = 4. We assume there are two common components in the spatial, spectral and temporal modes between two tensors, i.e., *L*_1_ = *L*_2_ = *L*_3_ = 2 and *L*_4_ = 0, which are parallel to the assumptions in the following ongoing EEG data processing. The spatial patterns were generated based on the brain activations located in the occipito-parietal, center, right occipital, frontal and left occipital regions (the 1st row of Figure 1A), respectively, corresponding to the frequency fluctuations centered at 10, 13, 20, 7, and 24 Hz (the 2nd row of Figure 1A). The temporal patterns were constructed using the time courses from the benchmark simulated complex fMRI dataset^1^ (the 3rd row of Figure 1A). The magnitude of participants is uniformly distributed, but for the common components, we limited the corresponding magnitude to (0, 0.2) and (0.5, 0.7) in order to better discriminate the two groups (the 4th row of Figure 1A).

We applied NCTF-ADMM algorithm to the simulated HC and MDD data, and the extracted spatial, spectral, temporal and participant components can be seen in Figure 1B. We conducted the algorithm 50 times, and got the averaged tensor fit of 0.9343 with the averaged running time of 111.85 s. The averaged correlation between the two sets of true factor matrices and recovered factor matrices is close to 1.

### 3.2. EEG Results

In terms of the number of components for the tensor data, a simple explained variance-based principal component analysis (PCA) method was adopted (Liu et al., 2021), and the number of principal components with 99% accumulated explained variance were assigned to *R*_HC_ and *R*_MDD_, then we set *R*_HC_ = 26, *R*_MDD_ = 36. Regarding the number of coupled components, we first ran the NCTF-ADMM without coupling constraints 10 times, and then we directly calculated the correlations in the spectral/spatial modes and performed correlation analysis in the temporal mode between the two groups of data, respectively. Finally, we selected the averaged number of highly correlated (0.87 and 0.90) and significantly correlated (*p* < 0.05) components as the number of shared components, i.e., *L*_1_ = *L*_2_ = *L*_3_ = 17 and *L*_4_ = 0.

**Figure 2:**
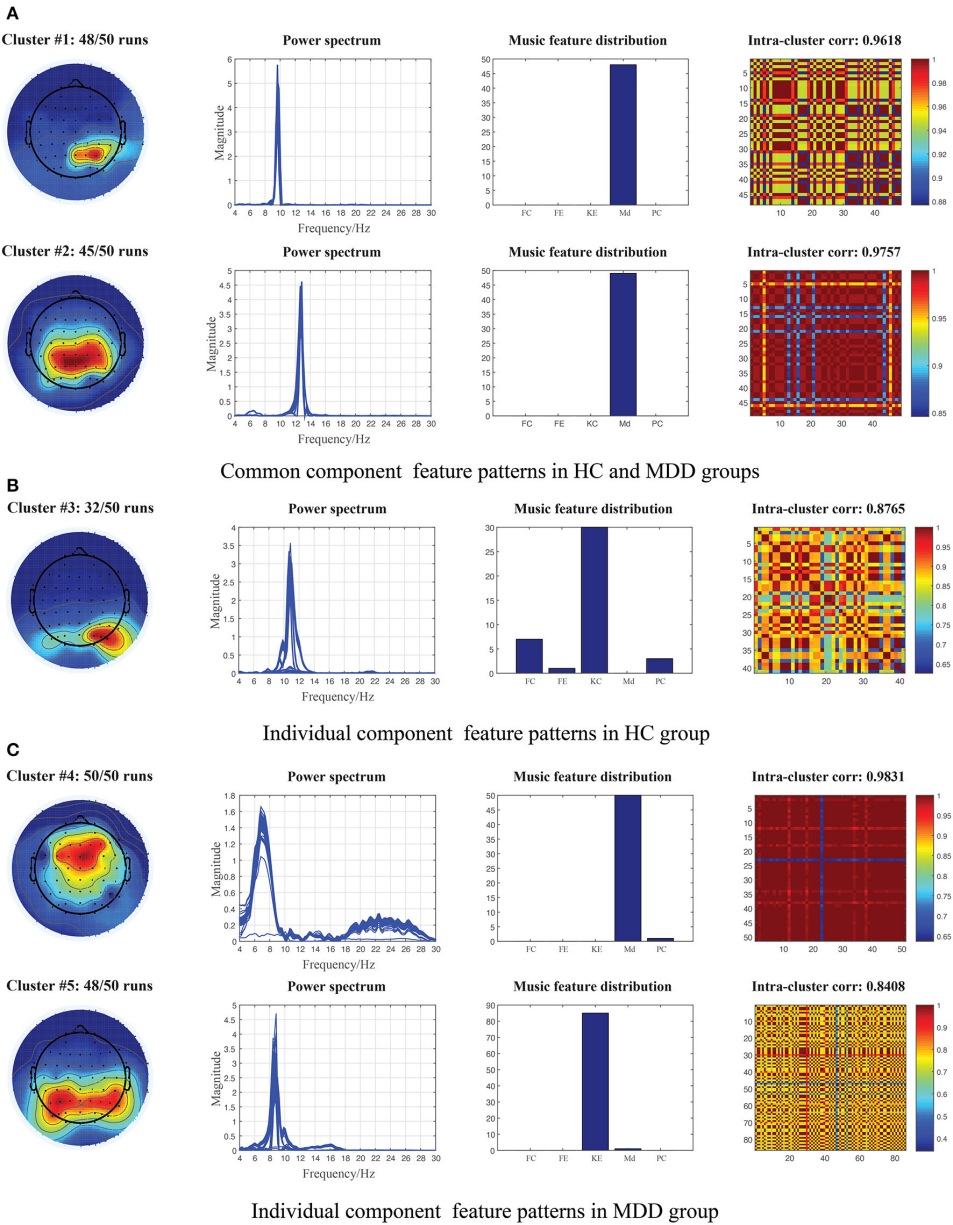
Illustration of the extracted component patterns (from left to right column: mean topography, overall power spectrum, music feature distribution and intra-cluster correlation maps) from HC and MDD EEG data via 50 runs of constrained tensor factorization method, and the parallel temporal component was significantly correlated with at least one of the musical features. **(A)** Common component patterns clustered from the shared components of 50 runs between HC and MDD data. **(B)** Individual component patterns clustered from the individual components of 50 runs in HC data. **(C)** Individual component patterns clustered from the individual components of 50 runs in MDD data. Md, Mode; KE, Key Clarity; FC, Fluctuation Centroid; FE, Fluctuation entropy; PC, Pulse Clarity.

We first carried out the proposed NCTF-ADMM algorithm 50 times on the two groups of ongoing EEG data, and then through correlation analysis and hierarchical clustering, we totally obtained 5 clusters of shared and unshared component patterns between/in HC and MDD groups. The averaged topographies, power spectrum, musical feature distribution and spatial correlation maps in the same cluster are plotted in Figure 2. Specifically, from the shared components extracted from HC and MDD data, we found two clusters of interested component patterns which were considered to be activated by the music modulation, and the probabilities of components in clusters #1 and #2 occurring in 50 runs reach 96% and 90%. Regarding cluster #1, the topography reveals that the right parietal region of the brain was activated with the low alpha oscillations modulated by the music feature “Mode”, while the cluster #2 represents the activation of parietal region of the brain with high alpha oscillations. The averaged correlations of spatial components in clusters #1 and #2 reached 0.9618 and 0.9757. For unshared components in HC group, we obtained one cluster in which the left occipital region was activated with the alpha oscillations and mostly modulated by the music features “Key Clarity” and “Fluctuation Centroid”. This cluster was unstable probably due to the low signal-to-noise ratio nature of EEG signals, and it was only clustered 32 times out of 50 times with the averaged spatial correlation of 0.8765. Moreover, the shared components in the MDD group were included into two clusters, cluster #4 reveals that the frontal region of the brain was activated with theta oscillations and modulated by the music feature “Mode”, and cluster #5 reveals that the modulation of musical feature “Key Clarity” brought about the activation of alpha oscillations in the parietal-occipital region of the brain. The occurrence probabilities of spatial components from clusters #4 and #5 in 50 runs are 100% and 96% with the averaged spatial correlations of 0.9831 and 0.8408.

## 4. Discussion

In this study, we investigated the shared and unshared brain activities of spatio-temporal-spectral modes from the HC and MDD data using EEG collections during freely listening to music. To this end, we proposed a complete solution combining constrained tensor factorization, musical information retrieval and spatial clustering, in which the brain activities of interest that were believed to be activated by music modulation were discovered. Through the time-frequency representation, we constructed two fourth-order tensors of time × frequency × space × participant for HC and MDD groups, and the two tensors were decomposed at the same time with the extraction of common and individual components using nonnegative coupled tensor factorization, and its performance has been verified using simulation data in section 3.1. Meanwhile, five long-term musical features, including two tonal and three rhythmic features, were extracted using the MIR toolbox. Then we adopted the correlation analysis to identify the components of interest whose temporal components were significantly correlated with any of the five music features, and performed clustering analysis on the outcomes of correlation analysis of the repeated runs in order to obtain reliable and convincing results.

For the simulated data, as shown in Figure 1, we can see that the simulated factor matrices representing space, frequency, time and participant loadings were successfully recovered using nonnegative and coupled tensor factorization with high tensor fittings. The participant loadings of the two coupled components are significantly different in the magnitude distribution. Compared with the conventional tensor factorization (Cichocki et al., 2015; Cong et al., 2015; Sidiropoulos et al., 2017), the constrained tensor factorization applied in this study simultaneously considered the shared and unshared information between/in the two groups of data via imposing the coupling structure (Zhou et al., 2016; Wang et al., 2019, 2020). Moreover, it can achieve unique solutions and interpretable components, while circumventing the independence constraint compared to its matrix counterparts (Calhoun et al., 2009; Hunyadi et al., 2017; Labounek et al., 2018; Zhu et al., 2021). As we all know, EEG signals are recorded as the multi-way tensors of time, space, subject, or group modes, thus multi-way analysis methods are attractive and promising tools for processing such tensor data, while the two-way analysis will lose the multilinear structure and hidden internal relationships in the original data (Cong et al., 2015).

The researchers have reported that a considerable amount of neural dynamic changes distributed in multiple subcortical and cortical regions (such as auditory, tactile, visual) were found in EEG/fMRI recordings during music perception (Andrade and Bhattacharya, 2003; Khalfa et al., 2005; King et al., 2019). These regions including hippocampus, hypothalamus, orbitofrontal and ventral medial prefrontal cortex are typically involved during emotion evocation, processing and hedonic regulation in voices (Menon and Levitin, 2005). Therefore, the potential of music to modulate activities in brain networks is worth investigating in MDD. Using the constrained tensor factorization-based framework, we were able to provide the feasibility to identify the brain dynamics involved in the processing of acoustical music from the ongoing EEG signals, and finally obtained two shared and three unshared feature patterns between or in HC and MDD data as shown in Figure 2. Studies have found that music can evoke a variety of emotions from simple arousal responses, basis emotions to more complex emotions such as subjective feeling, emotional expression or physiological changes in listeners (Witvliet and Vrana, 2007; Juslin et al., 2015). First, from the shared components extracted from both HC and MDD data, we obtained two clusters of interest patterns including a series of ~10 Hz right parietal components and ~13 Hz centro-parietal components, which were believed to be elicited by the tonal music feature of “Mode”. As we all know, MDD is a kind of mental disorders characterized by affective and cognitive dysfunctions, and existing studies have shown that brain networks of MDD patients have abnormal network topology structure (Gotlib and Joormann, 2010; Jia et al., 2010; Mulders et al., 2015). Moreover, the study also reported individuals with MDD were associated with impaired recognition of emotion in music as well as in facial and vocal stimuli (Naranjo et al., 2011). Therefore, in addition to some basic emotional processing and regulation that are indistinguishable from the MDD patients and the HCs, some uncontrolled responses with music of MDD patients may be more negative due to their cognitive dysfunctions. We also observed the right occipital components of ~10 Hz oscillations mostly elicited by the tonal feature of “Key clarity” and the rhythmic feature “Fluctuation Centroid” which were clustered from the individual components of HC group, but such brain dynamics in MDD patients were not sensitive to music perception and were suppressed. The dopaminergic system is activated during music processing, however, some dopamine responses to music in MDD patients may be weakened, which makes some brain neural dynamics that should be appeared not captured (Menon and Levitin, 2005; Blum et al., 2010). Our findings replicate some of the results of our previous studies in which similar alpha brain oscillations located in the centro-parietal or occipital regions were found from the EEG signals of 14 healthy participants when freely listening to music (Cong et al., 2013; Wang et al., 2020). Lin et al. also found the electrodes of parietal lobes across alpha band contributed a lot in the emotion recognition during music listening (Lin et al., 2010). Second, for the individual components extracted from MDD patients, we obtained two clusters of interest: theta oscillations in the frontal region and alpha oscillations in the bilateral parieto-occipital region, which were considered more overactive than the HC groups. From the perspective of functional connectivity in the source level, Liu et al. revealed three oscillatory hyperconnectivity networks including right hemisphere of alpha and beta bands, left auditory region of delta band and prefrontal region of delta band in MDD (Liu et al., 2021). The frontal region was involved in planning complex cognitive behaviour, decision making and working memory (Liu et al., 2021). In other words, the frontal regions played an important role in depression development and have received widespread attention (Rajkowska et al., 1999). Previous studies reported that the fronto-limbic neural networks were implicated in MDD, particularly in relation to the subgenual anterior cingulate cortex (ACC) which was considered to regulate amygdala activity in order to prevent excessive emotional reactivity and stress responses (Drevets et al., 1997; Phillips et al., 2003). The fMRI studies also uncovered the modification of functions in frontal and temporal regions (Wang et al., 2012). The alpha temporo-occipital component located in the left angular gyrus was engaged in music perception from most MDD patients (Zhu et al., 2021). Significant alternations of brain dynamics in the left frontal lobe, (left) parieto-occipital lob in theta and alpha bands were observed from the functional networks of MDD patients when using resting-state EEG signals (Sun et al., 2019; Zhang et al., 2020). The abnormal regions near parieto-occipital sulcus in MDD may be associated with the inability to detach from the visual dorsal stream, robust biases in attention or inhibitory control of irrelevant sensory (Gotlib and Joormann, 2010; Sacchet et al., 2016). Meanwhile, the sources in parieto-occipital regions were considered to contribute to the working memory load in the alpha band (Tuladhar et al., 2007). Our results are indeed consistent with some of the research findings in MDD, but in the end, it is difficult to compare directly due to the different methodological approaches and selected participants.

In conclusion, we provide a comprehensive framework for the shared and unshared feature extraction from the EEG recordings of MDD and HC groups during music listening, our findings are well supported and in line with the results of some previous studies to some extent, and contribute to providing some novel biomarkers for the clinical diagnosis and treatment of MDD patients. Meanwhile, the proposed methods based on nonnegative coupled tensor factorization may provide a new perspective for the analysis of other EEG recordings or the data with other psychiatric disorders. However, there are still some limitations in this study. First, we directly assume the temporal, spatial and spectral consistency among participants in MDD or HC group; that is, we only pay attention to group similarities and differences between the MDD and HC groups, and ignore the participant differences in each individual group, which will be a key issue that needs to be considered in our future work. Second, the analysis in this study was performed at the sensor level, which will be extended to the source level in the following work. Third, we have proposed a way to select the number of common components by correlation analysis. However, its selection is still subjective to some extent, which remains an open issue and invites more discussion.

## Data Availability Statement

The raw data supporting the conclusions of this article will be made available by the authors, without undue reservation.

## Ethics Statement

The studies involving human participants were reviewed and approved by First Affiliated Hospital of Dalian Medical University and Dalian University of Technology. The patients/participants provided their written informed consent to participate in this study.

## Author Contributions

XLW conceived and carried out the idea of the study, and drafted the manuscript under the guidance of JW and FC throughout the preparation process. WL took part in the introduction and discussion parts and provided critical revisions and feedback with the rest of authors. XYW preprocessed the data and revised the manuscript. ZM designed the experiment and EEG collected the data. JX and YC provided the EEG data used in this study, and QZ reviewed the manuscript. All authors contributed to the article and approved the submitted version.

## Funding

This work is supported by National Natural Science Foundation of China (grant no. 91748105), National Foundation in China (no. JCKY2019110B009 and 2020-JCJQ-JJ-252), the Fundamental Research Funds for the Central Universities (DUT2019 and DUT20LAB303) in Dalian University of Technology in China, Dalian Science and Technology Innovation Fund Project (2021JJ12SN38), and the scholarship from China scholarship Council (no. 201706060263).

## Conflict of Interest

The authors declare that the research was conducted in the absence of any commercial or financial relationships that could be construed as a potential conflict of interest.

## Publisher's Note

All claims expressed in this article are solely those of the authors and do not necessarily represent those of their affiliated organizations, or those of the publisher, the editors and the reviewers. Any product that may be evaluated in this article, or claim that may be made by its manufacturer, is not guaranteed or endorsed by the publisher.

## Footnotes

^1^ http://mlsp.umbc.edu/simulated_complex_fmri_data.html
